# An Automated Approach for General Movement Assessment: A Pilot Study

**DOI:** 10.3389/fped.2021.720502

**Published:** 2021-08-25

**Authors:** Camilla Fontana, Valeria Ottaviani, Chiara Veneroni, Sofia E. Sforza, Nicola Pesenti, Fabio Mosca, Odoardo Picciolini, Monica Fumagalli, Raffaele L. Dellacà

**Affiliations:** ^1^Department of Clinical Sciences and Community Health, University of Milan, Milan, Italy; ^2^TechRes Lab, Dipartimento di Elettronica, Informazione e Bioingegneria, Politecnico di Milano University, Milan, Italy; ^3^Fondazione IRCCS Ca' Granda Ospedale Maggiore Policlinico, NICU, Milan, Italy; ^4^Department of Statistics and Quantitative Methods, Division of Biostatistics, Epidemiology and Public Health, University of Milano-Bicocca, Milan, Italy; ^5^Fondazione IRCCS Ca' Granda Ospedale Maggiore Policlinico Milano-Pediatric Physical Medicine and Rehabilitation Unit, Milan, Italy

**Keywords:** general movement assessment, neurodevelopment, accelerometer, infant, newborn

## Abstract

**Objective:** The objective of the study was to develop an automatic quantitative approach to identify infants with abnormal movements of the limbs at term equivalent age (TEA) compared with general movement assessment (GMA).

**Methods:** GMA was performed at TEA by a trained operator in neonates with neurological risk. GMs were classified as normal (N) or abnormal (Ab), which included poor repertoire and cramped synchronized movements. The signals from four micro-accelerometers placed on all limbs were recorded for 10 min simultaneously. A global index (KC_index), quantifying the characteristics of individual limb movements and the coordination among the limbs, was obtained by adding normalized kurtosis of the distribution of the first principal component of the acceleration signals to the cross-correlation of the jerk for the upper and lower limbs.

**Results:** Sixty-eight infants were studied. A KC_index cut-off of 201.5 (95% CI: 199.9–205.0) provided specificity = 0.86 and sensitivity = 0.88 in identifying infants with Ab movements.

**Conclusions:** KC_index provides an automatic and quantitative measure that may allow the identification of infants who require further neurological evaluation.

## Introduction

Neurodevelopment is highly interconnected, especially in early infancy, where motor experiences drive cognitive and socio-emotional development (1, 2); in particular, in the first months of life, the presence of spontaneous movement leads to more directed and intentional movements through exploration and problem solving (3). Consequently, the characteristics of spontaneous movement in early infancy is a higher predictor for later neurodevelopmental performances (4). The identification of infants at high risk of adverse neurodevelopmental outcomes, including, but not limited to, cerebral palsy, is of paramount importance when planning specific intervention strategies (5), considering that the earlier the treatment, the higher the beneficial impact on the neurodevelopment of the child (6, 7). Early detection of infants at higher neurodevelopmental risk usually relies on the comprehensive evaluation of the clinical history, neuroimaging data, and different neuro-sensorial assessments (5, 7). Based on the clinical history, the American Academy of Pediatrics (8) suggests that preterm infants [especially those born ≤32nd week of gestational age (GA) or with a birth weight of ≤1,500 g] and term infants suffering from hypoxic–ischemic encephalopathy or other acquired perinatal brain lesions should be included in a neurodevelopmental follow-up program.

Among clinical neurological assessments, one of the most commonly used and reliable tools to detect early neurodevelopmental disorders is the Prechtl General Movements (GMs) assessment (GMA). GMs are spontaneous movements observable in infants up to the fifth month post-term, involving the entire body in a variable sequence and with different patterns of amplitude, intensity, and speed. In typical development, GMs are characterized by large movement variation, while a limited variation could indicate a higher risk for later neurodevelopmental impairments (9). GMs occur in age-specific patterns, in particular, from term equivalent age (TEA) up to the second month after term; the typical pattern is represented by the so-called “Writhing movements” (WMs), which are movements of the trunk and the four limbs with a small-moderate amplitude and moderate speed.

During the WMs period, abnormal GMs are classified as (a) poor repertoire (PR—repetitive sequences of movements, with low variability), (b) cramped synchronized (CS—rigid and not smooth nor fluent movements characterized by the simultaneous contraction and relaxation of all body parts), and, very rarely, (c) chaotic movements (Ch—abrupt movements with large amplitude and high speed). GMA, especially in those infants that exhibit early CS movements, provides high accuracy in the early identification of children at risk of developing cerebral palsy (9–11). The significance of PR movements has also been investigated and proven to be associated with the risk of later minor neurodevelopmental disorders that can affect the motor, cognitive, or socio-emotional areas (1, 12, 13). In particular, increasing evidence highlights how the persistence of a PR pattern after term is associated with a moderate to severe cognitive delay (14). In addition, Einspieler et al. described the presence of any abnormal GM pattern during the WM period with a later diagnosis of autism spectrum disorder or Rett syndrome (13). From 3 to 5 months post-term, fidgety movements (FMs) are present and are characterized by tiny movements of moderate speed and variable acceleration involving the neck, trunk, and limbs in all directions. The absence of FMs is highly predictive for the presence of cerebral palsy (15, 16).

GMA is performed through direct or video-recorded observation by a specifically trained operator, resulting in a qualitative description of the motor performance of the infants. However, the need for a trained examiner to accurately classify GMs over the observation period limits its widespread use, highlighting the importance of developing an objective and automated quantification approach. Recently, a growing interest in the development of an objective and measurable methodology has risen, and movement analysis has been performed according to different methods based on computerized approaches ranging from camera-based techniques to body-worn sensors (17). Most of the recent studies focused on camera-based techniques as they offer the advantage of not being in contact with the infant (18). The most used approach consists of 2D cameras combined with machine learning methods. However, machine learning methods require a large dataset for training as they are able to correctly classify only patterns they were trained on, and their behavior on different patterns is unpredictable (19). Using depth cameras, recent studies showed that it is possible to extract 3D trajectories (20). This method allows the computation of parameters that accurately describe movements as sensor-based methods can do. However, further development and validation of the method is needed as it requires very high computational power, the storage of a large amount of data, and the manual intervention of a technical expert (21, 22). Multiple wearable sensors have been developed to measure movements of infants, as reported in the review by Chen et al. (23): inertial measurement units (accelerometers and magnets), pressure sensors, and flexible sensors. Accelerometers and magnetic sensors are inexpensive, small, and lightweight. They provide robust real-time accurate quantitative data for movement evaluatuion (23), but a validated and clinically meaningful, standardized quantitative approach for data processing is still lacking.

Using clinical GMA as a reference, the present study aimed to develop an automatic quantitative approach to identify infants with abnormal limb movements at TEA, and to provide a simple and interpretable quantitative index to identify infants who would require further neurodevelopmental assessment.

## Materials and Methods

### Study Population

The study was carried out in the NICU of Fondazione IRCCS Ca' Granda Ospedale Maggiore Policlinico of Milan, Italy. All neonates considered at high neurodevelopmental risk who underwent at least one cranial ultrasound (cUS) according to the local clinical protocols were considered eligible for this pilot study. Neurodevelopmental risk was defined as the presence of at least one of the following criteria: preterm birth (≤32nd week of gestational age—GA), very low birth weight (≤ 1,500 g), or severe brain damage detected by cUS [defined as intracranial hemorrhage, including high grade−3 to 4—intraventricular hemorrhage (24), post-hemorrhagic ventricular dilation, extensive white matter damage—including cystic periventricular leukomalacia, cortical damage, and cerebellar hemorrhage].

Clinically stable neonates were evaluated at TEA in an alert behavioral state, at least 2 h from feeding. In the present pilot study, to recruit an adequate number of infants with abnormal GMs, priority was given to enrollment of infants with the highest neurological risk, identified as those with pathological cUS findings or abnormal neurological examination during NICU stay. Exclusion criteria were the presence of congenital malformations or genetic syndromes, ongoing mechanical ventilation, or the need for pharmacological sedation at TEA. The ethical committee of the Milano Area B (127_2015) approved the protocol, and parental written informed consent was obtained before entering the study. Characteristics of the study population were collected from the electronic hospital charts and included gender, birth weight, GA, small for gestational age (SGA) (25), twin birth, mode of delivery, type, and severity of brain damage.

### Study Protocol

**Figure 1:**
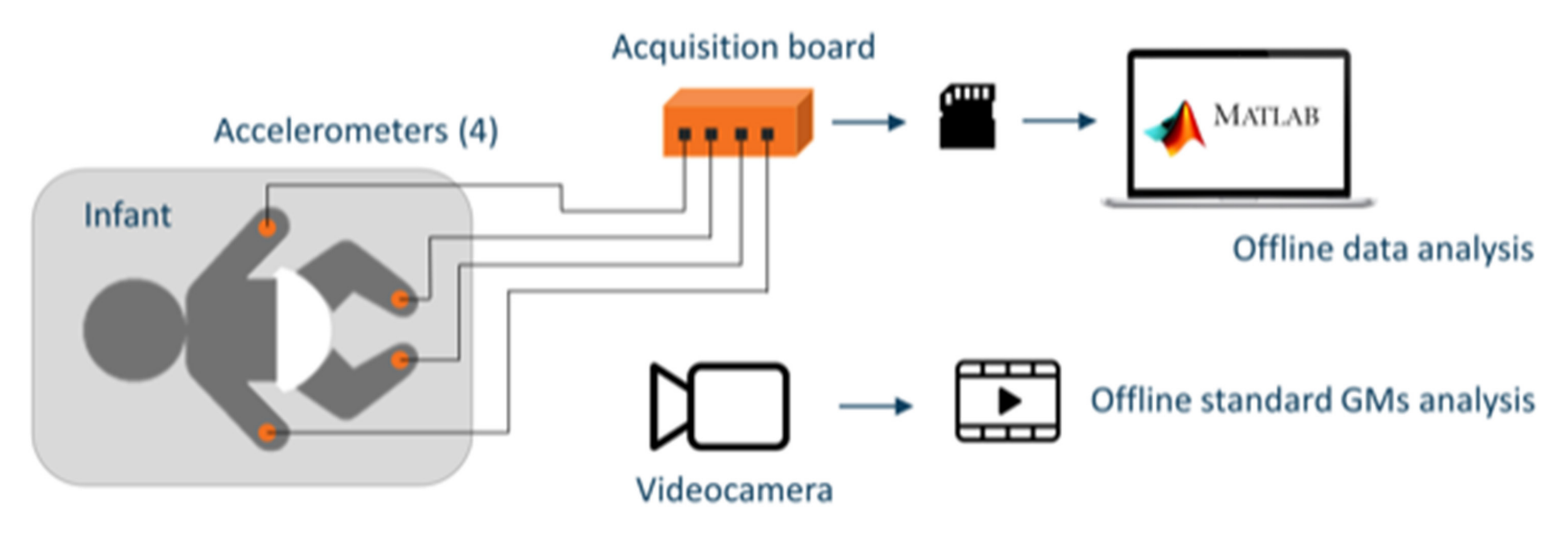
Schematic representation of the setup including the four accelerometers placed on the wrists and ankles of the infants and connected to the acquisition board that stores data on an SD memory card for offline analysis and the video recording device used for offline general movement assessment (GMA).

All participants simultaneously underwent the traditional GMA and accelerometer recording. Infants were assessed supine, only in a condition of clinical stability and in an alert behavioral state. Four three-axis micro-accelerometers

$$\begin{array}{l} KC\text{\_}index\;\left(i\right)=\;\left(\sum_{l=1}^{4}\frac{{kur\left(i\right)}_{l}}{max\left({kur}_{l}\right)-min\left({kur}_{l}\right)\;}+\;\sum_{p=1}^{2}\frac{{xcorr\left(i\right)}_{p}}{max\left({xcorr}_{p}\right)-min\left({xcorr}_{p}\right)\;}\right)*100 \end{array}$$

(LIS3LV02DL, STMicroelectronics, Switzerland) were placed on the lateral side of the ankles and the dorsal surface of the wrists. Thanks to their small dimension (4.4 × 7.5 × 1 mm and 72 μg), devices were secured with an elastic self-adhesive band (Figure 1). The recordings were started ~30 s after applying the sensors to let the infant regain an adequate behavioral state after being touched and to guarantee that the infant was no longer disturbed by the interaction with the operator. The acceleration signals were recorded on flash memory at a sampling frequency of 150 Hz for 10 min. A video recording allowed offline standard GMA; each recording lasted about 10 min, and to be included, it had to comprise at least five general movement sequences. To obtain the synchronization of the accelerometer signals, the start of acquisition was given contemporaneously to all the devices. To compensate for the small difference among the actual sampling rate of the four accelerometers (each accelerometer has its own timer), the actual sampling time of each data sampled by each accelerometer was saved and differences (<1 ms) compensated for.

### Data Analysis

Video and acceleration signals were analyzed independently by two different investigators. GMs of patients were classified by a trained operator as normal (N), poor repertoire (PR), and cramped synchronized (CS), according to the definitions by Einspieler et al. (9). In order to identify all infants at increased risk for later neurodevelopmental delay, we consider both PR and CS patterns as abnormal (Ab) as both are characterized by reduced complexity, variability, and fluency.

Acceleration signals were processed in MATLAB (The MathWorks, Inc., United States). All acceleration signals were high-pass filtered (cutoff frequency 0.05 Hz) to remove the gravitational component. As GMA includes both characteristics of single-limb movement pattern and coordination among limbs, we defined indexes to quantify both of them. For each limb, the principal components of the measured acceleration signal were computed. We considered the first principal component and used the kurtosis of the probability distribution, which represents the width of the probability distribution bell, to quantify the variability of movements of each limb. In particular, lower kurtosis values indicate less variability in the movements (narrower probability distribution), while higher values indicate high variability (wider probability distribution). The cross-correlation of the jerk (i.e., the rate of acceleration change) for upper limbs and lower limbs quantified movement correlation among the limbs. We normalized the computed values of each kurtosis and the cross-correlation by dividing their values for the difference between maximal and minimal values measured in all the subjects to provide a score between 0 and 1 for all the parameters. After normalization, the four kurtosis and the two cross-correlation measures were combined together into a new index (KC_index):

where *i* is the ith infant, *l* is the single limb, and *p* is for upper or lower limbs (hands, feet).

### Statistical Analysis

Baseline characteristics of the patients are reported as mean [standard deviation (SD)], median (range), and number (percentage), as appropriate. Independent *t*-test, Mann–Whitney *U*-test, and Fisher's exact test were used for comparisons between the groups.

The association between the KC_index and the clinical GM pattern was investigated using one-way ANOVA with Tukey HSD *post-hoc* test. Comparison between normal and abnormal groups was evaluated using a logistic regression model and odds ratio (OR) with 95% confidence interval (CI). The discriminatory power of KC_index to identify infants with abnormal movement patterns was assessed by the receiver operating characteristic (ROC) curve and the area under the curve (AUC), along with sensitivity and specificity measures, are reported. The optimal cutoff was chosen considering Youden's index. To evaluate the cutoff reliability, we used bootstrap methods (26). Bootstrap is a technique that allows estimating sample variability by drawing a large number of repeated samples from the original data and using them to build confidence intervals. In particular, we have drawn 1,000 samples with replacement of the same size of the original data, and we have used them to build the cutoff confidence interval.

The normal distribution of data was assessed using the Shapiro–Wilk test. All tests were two-tailed, and values of *p* < 0.05 were considered to be significant.

Considering an alpha level of 0.05, an 80% power, a 1.5 ratio between negative and positive cases, and an area under the ROC curve of 0.75 as significant, we estimated for this pilot study a minimum sample size of 65 subjects. Including a 15% dropout due to technical or clinical issues, the final sample size for the study was 77 subjects. All statistical analyses were performed using R version 3.5.1 (R Foundation for Statistical Computing, Austria).

## Results

A total of 77 infants were enrolled. Nine infants were excluded: seven infants because of non-optimal behavioral state during GM recording and two due to technical issues (i.e., detachment of one of the sensors) that prevented data recording. Infants were evaluated at a mean post-menstrual age (PMA) of 42.1 ± 2.4 weeks.

According to the clinical evaluation of GMs, 43 infants were categorized in the N group and 25 in the Ab group, with the latter including 17 PR and 8 CS. No chaotic GM pattern was observed. The N and Ab groups were comparable for baseline characteristics (Table 1), although infants in the Ab group were more likely affected by severe brain lesions (5 vs. 64%, *p* < 0.001) and had a longer hospital stay (51 vs. 73 days, *p* = 0.045).

**Table 1:** Baseline characteristics of the population presented for normal (N), poor repertoire (PR), cramped synchronized (CS), and abnormal (Ab) comprising both PR and CS.

	**N**	**PR**	**CS**	**Ab**	***P-value***
	**(*n* = 43)**	**(*n* = 17)**	**(*n* = 8)**	**(*n* = 25)**	**N vs. Ab**
Gestational age at birth (weeks), mean (sd)	30.8 (2.4)	31.2 (5.3)	32.8 (3.3)	31.7 (4.8)	0.378^a^
Birth weight (g), mean (sd)	1,357 (407)	1,596 (968)	1,769 (776)	1,651.3 (898)	0.132^a^
Female, *n* (%)	26 (60)	5 (29)	6 (75)	11 (44)	0.215^b^
Small for gestational age, *n* (%)	10 (23)	3 (18)	2 (25)	5 (20)	>0.999^b^
Twins, *n* (%)	28 (65)	7 (41)	4 (50)	11 (44)	0.127^b^
Cesarean section, *n* (%)	38 (88)	13 (76)	6 (75)	19 (76)	0.305^b^
Severe brain damage, *n* (%)	2 (5)	9 (53)	7 (88)	16 (64)	<0.001^b^
Length of NICU stay (days), median (range)	51 (14–104)	51 (11–168)	85 (26–127)	73 (11–168)	0.045^c^
Maternal age (years), mean (sd)	34 (5.1)	35.2 (6.1)	30.6 (5.1)	33.8 (6.1)	0.856^a^
Gestational age at GMs evaluation (weeks), mean (sd)	42.0 (2.5)	42.3 (2.6)	41.7 (1.2)	42.2 (2.2)	0.817^a^

*The p-value refers to comparisons between N and Ab*.

a *t-test*.

b *Fisher's exact test*.

c *Mann-Whitney U-test*.

**Figure 2:**
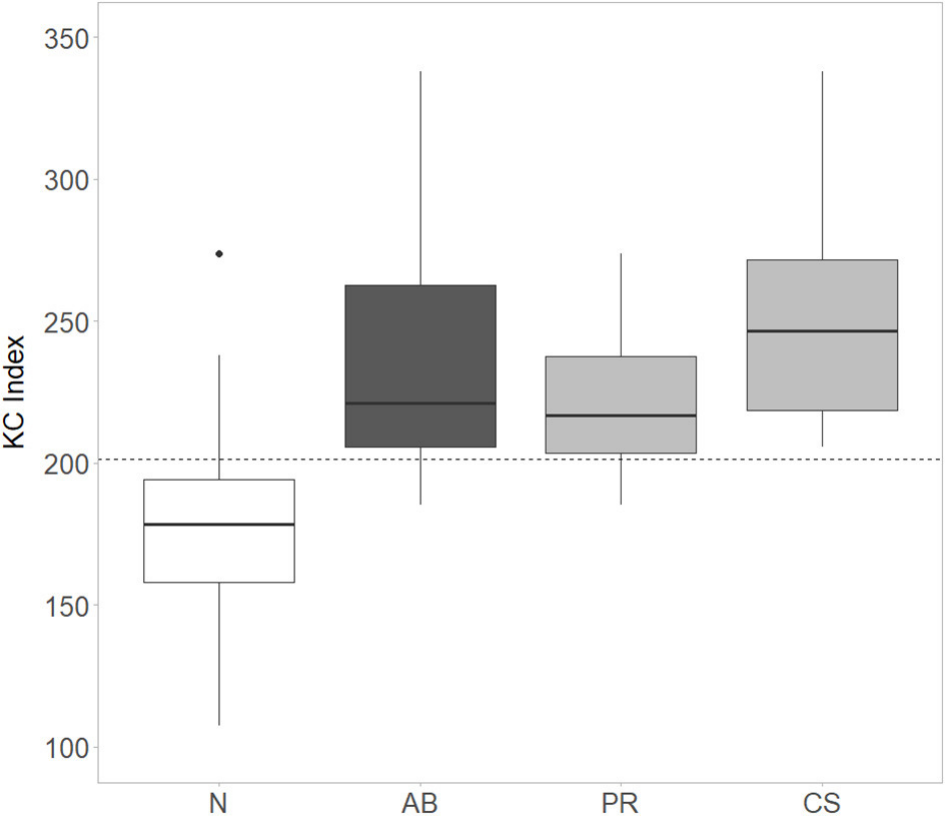
Distribution of the KC_Index for normal (N), abnormal (Ab = CS+PR), poor repertoire (PR), and cramped synchronized (CS) subjects. The identified KC_Index cutoff is indicated by the dashed line. Comparison between N, PR, and CS groups: *p* < 0.001. N vs. PR, *p* < 0.001; N vs. CS, *p* < 0.001; PR vs. CS, *p* = 0.095, ANOVA with Tukey's HSD *post-hoc* test.

**Figure 3:**
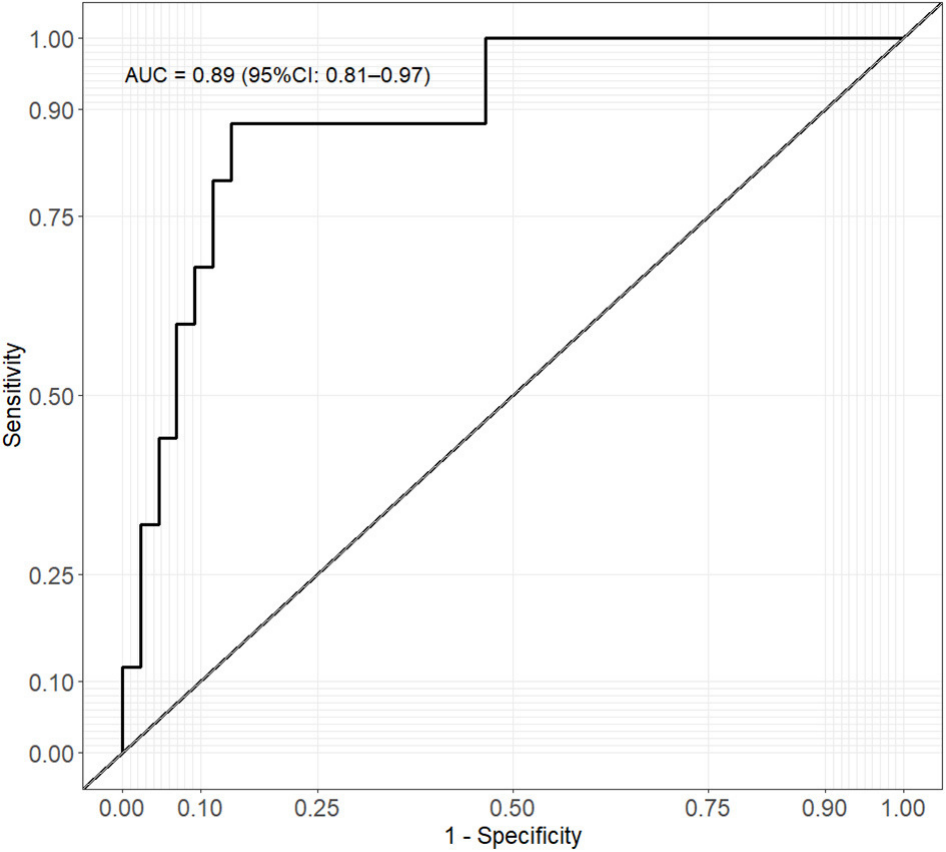
Receiver operating characteristic (ROC) curve analysis of KC_index and clinical GMA (normal vs. abnormal).

A positive association was observed for the KC_index and the clinical GMA, showing an increase in the index value to the worsening of the motor function. The KC_index mean (SD) was 179.2 (31.0), 223.8 (29.6), and 253.1 (44.1) for the N, PR, and CS groups, respectively (ANOVA *p* < 0.001) (Figure 2). This relationship was confirmed by logistic regression model estimate when comparing N vs. Ab groups (OR: 1.058, 95% CI: 1.032–1.094, *p* < 0.001). When a KC_index cutoff of 201.5 (95% bootstrap CI: 199.9–205.0) is used, the specificity in identifying Ab from N is 0.86, the sensitivity is 0.88, and a corresponding area under the ROC curve (AUC) is 0.89 (95% CI: 0.81–0.97) showing a clear association with the clinical GMA (Figure 3). Moreover, with the identified cutoff, all the CS patients were correctly classified in the abnormal group. Considering only the kurtosis or the cross-correlation, we obtained worst performances with an AUC of 0.80 and 0.72, and the 38% and 75% of the CS patients were correctly classified in the abnormal group, respectively.

## Discussion

The early identification of infants at high risk of adverse neurological outcomes (including cerebral palsy) is essential to promote targeted neurodevelopmental interventions, but it basically relies on the clinical assessment performed by highly specialized health care professionals. This study provides evidence that the use of accelerometers, together with an appropriate data analysis algorithm, can be used as an automatic tool capable of discriminating with high accuracy abnormal GMs at TEA.

Several methods for motion detection of GMA have been described; they can be divided into direct and indirect sensing methods (17, 27). Among the indirect methods, 2D RGB webcams are the most used (22), as they have the advantages of being easily available at low cost and are not in direct contact with the studied infant. However, with 2D image analysis, information related to the third dimension, which may represent up to 53% of the movement (21), is lost. They can also be affected by image occlusion and by errors in tracking limb position with time.

Differently direct methods, including accelerometer-based methods, allow high temporal resolution; they are low cost, robust to artifacts, and accurate in describing the movement. They also present better performances than webcam-based technologies for motion impairment prediction (22). Disadvantages of the accelerometer-based approach in comparison with the webcam-based approach include low spatial resolution and physical contact between the sensor and the infant. Although more intrusive compared with video cameras, the sensors we used were safe and well-tolerated by all the study participants. The interruption of few evaluations was mainly related to the changes in the behavioral state of the infant not dependent on the sensors themselves. This technology is easy to use; indeed, the four sensors were rapidly and easily applied on the wrists and ankles of the infant close to anatomical landmarks and easily removed and disinfected after the measurement. The sensors and the box containing the motherboard are light and practical, making the experimental setting suitable for any situation in which a soft flat surface is available. We experienced no data lost and long battery life (more than 12 h). The user friendliness of accelerometer-based devices is also demonstrated by the recent study by Prioreschi et al. (28) that developed a practical and portable wearable wrist band enclosing an accelerometer, easier to secure to the infant limbs and allowing free movements.

Even if webcam-based approaches provide higher spatial resolution, the use of four sensors at the same time enables the comparison and correlation of movements of different limbs, thus, obtaining information on simultaneous movements, those in the same direction, and those with a specific repetitive sequence. Previous studies analyzed patterns of a single-limb movement using only one or two accelerometers (29, 30), being less intrusive for the infants but, at the same time, less informative on the general movements of the infants.

Relative to the time of assessment, we considered for the analyses a standard time of 10 min of recording, which is a reasonable time span to observe several sequences of GMs and during which infants at TEA are likely to maintain a quiet alert state before behavioral changes occur. Very different recording durations have been reported by previous authors: Heinze et al. (31) performed evaluations lasting around 20 min in infants aged 1–5 months, while Ohgi et al. (29) only recorded movements for about 200 s at 1 month post-term age. The possible recording time depends on postnatal age as older infants show more stable behavioral status, allowing longer recording, compared with the younger ones (like in our case) in which the quiet state is short lasting; however, too short recordings, as the reported 200 s, may not be enough as few movement sequences can be captured in this short time frame and a limited interpretation can be given to the motility in general.

In our study, we combined all the abnormal GMA observed, merging CS and PR pattern. This decision was based on the primary aim of the study that is the development of an automatic quantitative approach to early and easily identify abnormal GMs deserving further investigations and not to distinguish between different movement patterns. In addition, several studies identified both CS and PR patterns as possible warning signs of later neurodevelopmental delay as demonstrated by the fact that almost up to half of the infants that display PR movements at TEA may have an abnormal fidgety (32) pattern or, later in life, may show a neurodevelopmental delay (33). We acknowledge that the combination of both PR and CS movements might limit the possibility to predict long-term outcomes; however, this was beyond the purpose of the present study.

Most of the previous studies considered accelerometer data for automating GMA are based on genetic algorithms or machine learning approaches (17, 31, 34–37). These are also almost the exclusive methods applied to 2D RGB camera data mimicking the clinical evaluation (38–42). Machine learning methods have great potentialities for classifying data. However, they require an extensive training set that encompasses all the possible motion characteristics of healthy and pathologic infants and provide black-box classifications without a clear physiologically based rationale. It is impossible to predict the model performances on patterns not included in the training set (19). To our knowledge, nowadays, large datasets are not available. Despite applying methods for mitigating model overfitting (multiple videos from the same infant, 10-fold random split cross-validation, leave-one-out cross-validation, and generation of synthetic data), the prediction models developed in previous studies are based on small populations (38–42) and can be highly dependent on the motion pattern of the few studied infants. A larger validated dataset is needed to allow further advancement in this automatic approach (18).

Different from previous studies, we propose an index designed for mimicking the clinical criteria used to classify motion patterns that combines information on single-limb movement patterns and coordination among limbs. We decided to compute together data from the upper and lower limbs as the classification of the general movements developed by Prechtl et al. rely on the evaluation of the movements of the whole body (9). We validated our approach over a relatively large population and in a closely resembling real-life clinical setting. With this preliminary study, we were able to identify an index that can be used as a more quantifiable measure of general movements, allowing the identification of different movement patterns. In addition, the reported threshold for the KC_index could help clinicians to define a more accurate evaluation on an individual basis and to identify those infants that require further neurologic assessments. We obtained a sensitivity of 0.88 and specificity of 0.86, and that are consistent with the best ones (0.71 and 0.83) reported for webcam-based approaches in a similar population (18). Further studies should address the prognostic value of this parameter in predicting long-term neurological deficits, especially in the case of PR pattern, which is known to be associated with a less predictable outcome.

The present study has some strengths and limitations. Relative to the methodology, one of the advantages is that the GMA was performed and evaluated by the same clinicians, reducing the inter-operator variability both in the preparation of the experimental setting and in the clinical interpretation of GMs. Moreover, the exact position of the four sensors was established at the beginning of the study and has never been changed. Nevertheless, the acceleration signal assessed only the movement of the distal portion of the four limbs, therefore, not including the movements of the trunk, which represents a limitation for the clinical movement analysis. A second limitation is related to the inclusion of a non-consecutive series of participants; however, the sample size included is larger compared with previous studies aimed at the GM quantification (17). Finally, considering unequal weights for the variables included in the KC_index may provide better results. However, we felt that our study population was smaller for allowing variable weights to be optimized and that the resulting weights would have been more dependent on the population enrolled. Therefore, we only normalized the variables for their range and used equal weights to give the same importance to variability in the movements and coordination of the limbs.

In conclusion, our results support that this approach provides an automatic and quantitative figure that may allow the early identification of infants with an abnormal movement pattern deserving a more accurate evaluation by dedicated trained personnel. The present study explores a novel approach to movement analysis, focusing on *a priori* defined parameters chosen according to the known specific characteristics of abnormal movements to better quantify the complex patterns of neonatal general movements. The method presented is objective, quantitative, and easy to apply; these are essential requirements to be fulfilled in order to make this technology clinically available for the evaluation of large numbers of infants. More extensive studies are needed to assess whether this automated approach can be properly used as a screening tool, which could be used by less experienced clinicians to distinguish between normal and abnormal general movements.

## Data Availability Statement

The original contributions presented in the study are included in the article/supplementary material, further inquiries can be directed to the corresponding author/s.

## Ethics Statement

The studies involving human participants were reviewed and approved by Ethical committee of Milano Area B (127_2015). Written informed consent to participate in this study was provided by the participants' legal guardian/next of kin.

## Author Contributions

CF, CV, OP, FM, MF, and RD conceived and designed the research. CF, VO, and SS performed the experiments. CF, VO, CV, OP, MF, and RD interpreted the results of the experiments. VO, NP, and CV analyzed the data. VO prepared the figures. CF, VO, and RD drafted the manuscript. CV, SS, NP, OP, FM, and MF edited and revised the manuscript. All authors contributed to the article and approved the submitted version.

## Conflict of Interest

The authors declare that the research was conducted in the absence of any commercial or financial relationships that could be construed as a potential conflict of interest.

## Publisher's Note

All claims expressed in this article are solely those of the authors and do not necessarily represent those of their affiliated organizations, or those of the publisher, the editors and the reviewers. Any product that may be evaluated in this article, or claim that may be made by its manufacturer, is not guaranteed or endorsed by the publisher.
